# Supplementary material for: Effects of ‘The Vicious Worm’ educational software on Taenia solium knowledge among key pork supply chain workers in Zambia
Source: PLoS Negl Trop Dis. 2020 Oct 19;14(10):e0008790. doi: 10.1371/journal.pntd.0008790 (PMC7595624; doi:10.1371/journal.pntd.0008790)
Supplement: S1 File — A list of questions asked during the ‘pre’, ‘post’ and ‘3weekspost’ questionnaire survey. (PDF) [file pntd.0008790.s002.pdf]

Vicious Worm Question list:

ID:____________________

1) **Have you ever heard about *masese/mase/mushokwe*?**
   A. No.
   B. Yes

2) **Can people get *masese/mase/mushokwe?***
   A. Yes
   B. No

3) **How can a pig become infected with *masese/mase/mushokwe*?**
   A. From dirt getting into wounds.
   B. From eating human stool.
   C. From eating moldy maize bran or rice.
   D. From being mated with an infected pig.
   E. I don't know.

4) **How can you test for *masese/mase/mushokwe* in a live pig?**
   A. By examination of the tongue.
   B. By feeling the belly.
   C. By checking the skin.
   D. You cannot test it in live pigs.
   E. I don't know.

5) **How can *masese/mase/mushokwe* be diagnosed in a slaughtered pig?**
   A. By inspecting the stomach and intestines for worms.
   B. By inspecting the lungs for black areas.
   C. By inspecting carcass and organs for white cysts.
   D. You cannot diagnose it in a slaughtered pig.
   E. I don't know.

6) **What has to be done with a slaughtered pig with porcine cysticercosis?**
   A. Nothing as the pig isn't contagious anymore.
   B. Destruction of the whole carcass.
   C. Cut out the infected part and sell what is left to prevent waste.
   D. I don't know.

7) What does *masese/mase/mushokwe* look like? ID:______________________

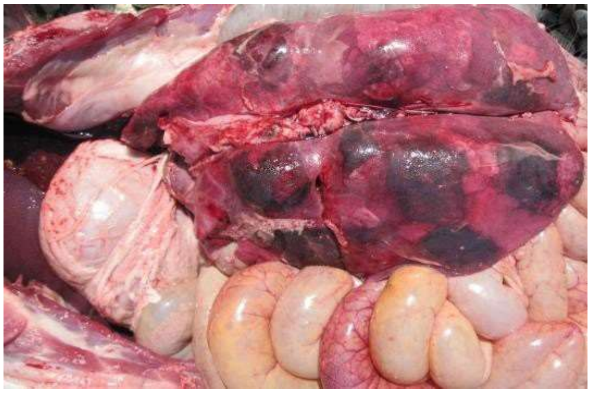

Picture A: black areas on the lungs

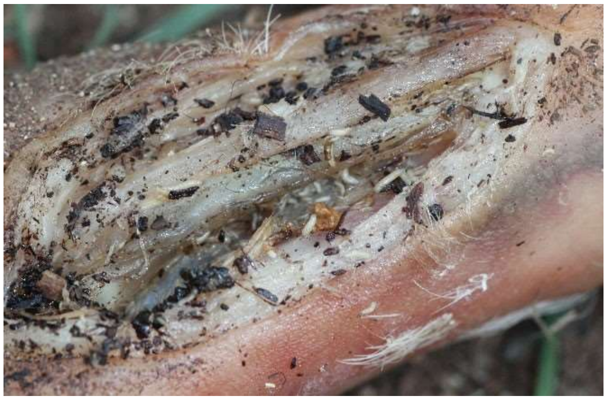

Picture B: wound on a pig foot

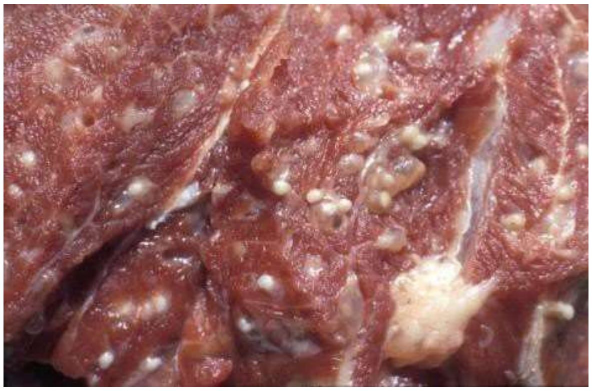

Picture C: White cysts in the muscles and organs

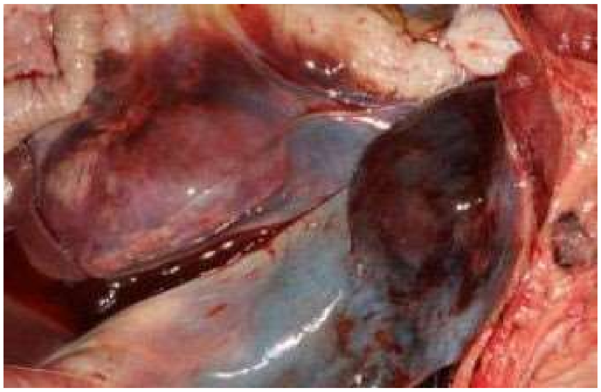

Picture D: Swollen kidneys

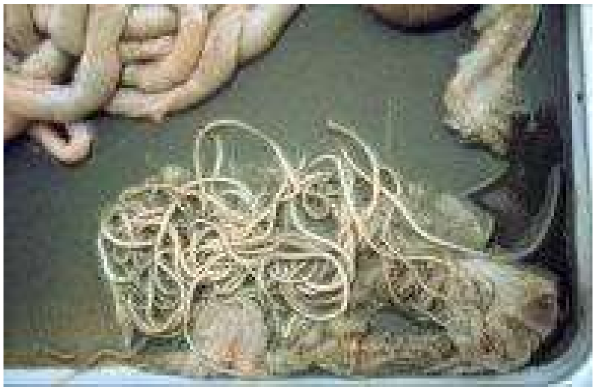

Picture E: Worms in the intestine

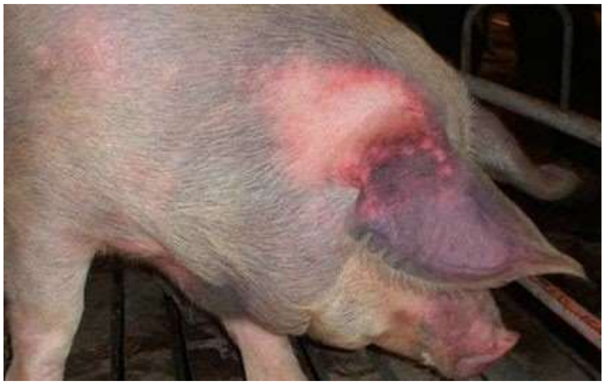

Picture F: Reddening of the ears

8) What should ideally be done with a live pig with *masese/mase/mushokwe*?

A. Treat the pig with ash.
B. Give the pig specific medicine.
C. Isolate the pig from the other pigs.
D. No treatment is needed before slaughter.
E. Kill the pig as it is contagious.
F. I don't know.

9) How can you prevent pigs from getting *masese/mase/mushokwe*? ID:______________________

A. Isolate the pigs that are infected.
B. Avoid pigs' access to human stool.
C. Don't feed moldy maize bran or rice.
D. Clean wounds regularly.
E. Prevent mating with infected pigs.
F. I don't know.

10) Are *masese/mase/mushokwe* and human tapeworm related?

A. Yes
B. No
C. I don't know

11) How do people get pork tapeworm infection?

A. By contact with pigs with infected wounds.
B. By contact with sick pigs or sick people.
C. By eating raw or undercooked infected pork.
D. I don't know.

12) What is a possible method to diagnose pork tapeworm infections in people?

A. It cannot be diagnosed.
B. By looking at a stool sample under a microscope.
C. By looking in the ear canal with a specialized tool.
D. By checking the skin for infected wounds or worms under the skin.
E. I don't know.

13) A person with a pork tapeworm will spread many tapeworm eggs through…

A. …coughing and sneezing.
B. … defecating.
C. … urinating.
D. …sharing drinks or kissing.
E. …open wounds or pus.
F. I don't know.

14) How can the pork tapeworm be treated in people?

A. It cannot be treated
B. By taking specific medicine.
C. By going to the traditional healer.
D. By only eating salted rice for 1 week.
E. I don't know.

15) How can you prevent the pork tapeworm in people?

A. By eating a tablespoon of salt a day.
B. By cooking pork so it is well done.
C. By regularly cleaning open wounds.
D. By avoiding physical contact with sick people.
E. By avoiding physical contact with sick pigs.
F. Do not know.

16) What is human neurocysticercosis? ID:____________________

A. It is a disease that can cause skin wounds.
B. It is a disease that can cause stomach cramps.
C. It is a disease that can cause fever.
D. It is a disease that can cause headaches and epilepsy.
E. I don't know.

17) A person with neurocysticercosis/cysticercosis may have got the infection by?

A. Eating vegetables contaminated with tapeworm eggs from human feces.
B. Eating undercooked pork infected with masese/mas/mushokwe .
C. Physical contact with a pig infected with masese/mase/mushokwe.
D. Physical contact with a person with fever
E. I don't know.

18) What should a person who experiences seizures or severe chronic headache do?

A. Seek assistance from the health center.
B. Be isolated from others.
C. Not share drinks with others.
D. Seek assistance from a traditional healer.
E. I don't know.

19) Can a person with neurocysticercosis transmit the disease to other people?

A. Yes, via infected stool.
B. Yes, via direct skin contact.
C. Yes, via coughing and sneezing.
D. Yes, via sharing drinks with others.
E. No, it cannot be transmitted.
F. I don't know.

20) Do you think meat inspection should be performed?

A. Yes, but only if the meat will be sold.
B. Yes, but only when the pig shows symptoms/is diseased.
C. Yes, but only when the pig owner wants it/asks for it.
D. Yes, always.
E. No, never.

21) Have you ever discovered porcine cysticercosis in a slaughtered pig?

A. Yes, often, at least once a week
B. Yes, regularly, but less than once a week
C. Yes, but very rarely, less than a few every year
D. No
E. I don't know.

22) What was done with the pig with porcine cysticercosis? (allow multiple options)

A. Nothing was done about it
B. The infected part of the pig was removed.
C. The pig was treated with ash or salt.
D. The pig was returned to the farmer
E. The whole carcass was destroyed
F. I don't know.
