# Supplementary material for: Effects of ‘The Vicious Worm’ educational software on Taenia solium knowledge among key pork supply chain workers in Zambia
Source: PLoS Negl Trop Dis. 2020 Oct 19;14(10):e0008790. doi: 10.1371/journal.pntd.0008790 (PMC7595624; doi:10.1371/journal.pntd.0008790)
Supplement: S2 File — Overview of key points that were discussed during the focus group discussions. (PDF) [file pntd.0008790.s003.pdf]

Key points FGDs

Table 1: Positive aspects of TVW

Group	Positive opinions about TVW	Specific positive aspects of TVW
Lusaka 1	Good format	- Simple/clear
	Educative (learned …)	- Bring patients to hospital<br>- Clinical signs NCC<br>- Hygiene (e.g. washing hands after toilet use)<br>- Importance of using toilets<br>- Pig husbandry<br>- Preparation of food (e.g. thoroughly cook pork, washing vegetables)<br>- Transmission and lifecycle
	Good illustration	
	Can be used to teach others	
Lusaka 2	Good format	- Simple/clear
	Learned something	- Bring patients to hospital<br>- Thoroughly cook pork<br>- Transmission and lifecycle
	Good illustration	
	Can be used to teach others	
Katete 1	Good format	- Simple/clear
	Educative (learned …)	- Danger of masese<br>- Importance of meat inspection<br>- Importance of using toilet<br>- Pig husbandry<br>- Transmission and lifecycle
	Can be used to teach others	
Katete 2	Good format	- Simple /clear
	Educative (learned …)	- Danger of masese<br>- Pig husbandry<br>- Preparation of food (e.g. thoroughly cook pork)<br>- Transmission and lifecycle<br>- Treatment human<br>- Treatment pig
	Good illustration	
	Can be used to teach others	

Table 2: Possible improvements to TVW

Group	Possible improvements to TVW	Specific possible improvements to TVW
Lusaka 1	Accessibility	- Electronics needed
	Adding videos	- Not cartoons
	Better explain acquisition HCC/NCC	- How the cyst gets into a human being, that part needs to be explained better.
	Larger scale	
	More languages	

Lusaka 2	Add videos	
	Clearly state the part that talks about masese causing seizures.	
	Larger scale	
Katete 1	Add videos	- Importance of use of toilets can be clearer with videos<br>- Not cartoons
	Larger scale	
Katete 2	Accessibility	- Electronics needed

Table 3: Alternative methods to disseminate knowledge

Group	Alternative methods to disseminate knowledge
Lusaka 1	Booklet
	Drama or sketches
	Flyer
	Radio
	Television
Lusaka 2	Drama
	Poem
	Handouts
	Flyers
	Radio
	Sending 'teachers' to villages
	Television
	Videos
Katete 1	Booklets
	Drama
	Radio
Katete 2	Booklet
	Community meetings through the headmen
	Drama or sketches
	Radio

Table 4: Assessment of the seizure videos

Group	Assessment	Specific comments
Lusaka 1	Unanimously agreed as being acceptable to use in TVW	- The videos help convey the message<br>- Videos help to raise awareness<br>- Would be a good idea to show these videos to the farmers who keep these pigs<br>- Shouldn't be limited to only farmers, the message concerns everyone
	Discussed concerns	- Videos could have negative impact on the pig business<br>- However, the public health issue outweighs the economic concerns

	Message	- Videos will be able to highlight the severity and importance of the problem<br>- People will know the importance of keeping pigs in good condition and might improve the way of slaughter<br>- Videos will change how people cook pork meat
Lusaka 2	One participant argued that these videos shouldn't be used.	- I think that the videos will lead to poor business as people will stop buying the pork.
	The majority of the participants found the videos to be appropriate	- Important for people to see how big the problem is<br>- The addition of videos could save lives (strong visual impression)<br>- Causal link should be made clear prior to display of video
	Message	- Videos will show villagers that the seizures have a scientific explanation (nothing supernatural)<br>- People will be more likely to seek medical help from hospitals instead of going to a traditional healer<br>- People will refrain from buying infected meat<br>- People will cook pork meat properly
Katete 1	Unanimously agreed as being acceptable to use in TVW	- The videos comprehensibly show the danger and gravity of the disease<br>- Will help people to make informed decisions regarding pork meat
	Discussed concerns	- Pig traders are begging not to show the videos for the sake of their businesses<br>- Their economic concerns aren't as important as the public health issue
	Message	- Videos help to recognize the disease<br>- Videos can be used to discourage people from eating infected meat<br>- The educated should take it upon themselves to teach others in the villages about the dangers of TVW<br>- Videos show the gravity of the problem and the consequences<br>- Can lead to adoption of control measures which in turn might bring down the number of masese cases
Katete 2	Unanimously agreed as being acceptable to use in TVW	- There is no problem whatsoever with showing these videos to inform the people about TVW
	Discussed concerns	- Videos might negatively impact the pig business<br>- The negative impact will only occur if the people (consumers) are not taught properly<br>- A participant argued that if not comprehensibly explained some people won't fully understand and will therefore blame pork meat as a whole for masese, which in turn would reduce the pig business<br>- Therefor a clear explanation of the cause of the seizures is crucial

	Message	- People will learn about the importance of toilets. It will also make people build and use toilets.<br>- The videos might incite people to keep their pigs enclosed

Table 5: Control Hurdles

Group	Control hurdle	Specific comments
Lusaka 1	Trade of infected pigs and/or pork	- Infected pork is cheaper, and a lot of people would rush for it without knowing the danger.<br>- There are people that specifically look for infected pigs. We have their phone numbers and call them when we find masese.
	Free-ranging pigs	- Instead of keeping pigs free ranging we need to keep them in a kraal.
	Maintaining a good relationship with the public	- Slaughterhouse workers would be accused of putting masese on the carcass using acid and a wire.
	Inadequate meat inspection	- The people from the veterinary department don't know about masese. When we slaughter, they don't even inspect the carcass.
	Inadequate sanitation	- We have seen when someone goes to the bush, the pig is waiting. You can get a stick, but it will not go away until it has eaten the feces.<br>- Open defecation happens in the communities and villages.
	Undercooked pork	- Sometimes we don't know how to cook pork properly, some just fry it fast and give it to their children without knowing that the disease is still in the meat.
	Unknown origin vegetables	- vegetables are grown in a lot of places that we don't know
	Lack of knowledge: HCC/NCC	- Because of lack of knowledge we would think that it was because of members of the family bewitching each other.<br>- My second borne used to fit and I have gone everywhere from one witch doctor to another, even went for prayers but nothing helped until I went to the hospital.<br>- Others say when you just touch someone who fits then you will get the disease.
	Lack of knowledge: PCC	- We thought masese was the result of feeding pigs residues from beer brewing.<br>- We didn't even know that there was a relationship between masese and pigs eating human feces.<br>- Farmers still think till think that what brings masese are the things they feed the pigs like beer residues.
	Lack of knowledge: Relation PCC-HCC-taeniosis	- We didn't even know that there was a relationship between masese and pigs eating human feces.
Lusaka 2	Trade of infected pigs and-or pork	- Infected pork continues being bought and consumed.<br>- We are being careless when buying pigs.
	Free-ranging pigs	- We are being careless about raising pigs.

	Lack of knowledge: HCC/NCC	- The video will show that the seizures are not because someone has been bewitched.<br>- When people see someone with seizures, they want to go to the traditional healer instead of going to a hospital.<br>- We used to think that when someone started having seizures it was because he was drunk.
	Lack of knowledge: PCC	- We used to think that masese is fat in the meat.<br>- We didn't know how a pig gets masese.<br>- We didn't even know that there was a tapeworm which brings about masese.
	Lack of knowledge: Relation PCC-HCC-taeniosis	- We learnt how this disease moves from humans to pigs and vice versa.<br>- We didn't know that there was a tapeworm which brings about masese.<br>- I didn't know that when you eat masese you can have the tapeworm; but now I know.
Katete 1	Trade of infected pigs and-or pork	- There are colleagues who buy pigs from villages without checking and even when they slaughter, they don't check but just sell the meat.
	Inadequate meat inspection	- There are butchers who buy pigs from villages without checking for masese. And even when they slaughter, they don't check but just sell the meat.
	Free-ranging pigs	- Pigs go to the bushes to eat the feces.
	Maintaining a good relationship with the public	- Sometimes when you kill a pig and find masese and you try to tell people not to eat, they don't understand and won't believe you.
	Inadequate sanitation	- People go to the bush to defaecate.
	Lack of knowledge: HCC/NCC	- In the villages everyone will just say that a person with epilepsy has been bewitched. Even we didn't know that masese can affect people, but now we have learned.
	Lack of knowledge: PCC	- There are many people in the villages that don't know that masese in pigs is a disease and that it can affect people.<br>- We would see masese but we did not know what it is and that's the reason why even when the meat was infected, we would just eat it.<br>- There are people like our parents who keep pigs and when these have seizures, they do not know what's happening so such videos will make them understand. Because they always say its ASF and yet it could be another disease.
	Lack of knowledge: Relation PCC-HCC-taeniosis	- There are many people in the villages that don't know that masese in pigs is a disease and that people can also get it.
Katete 2	Free-ranging pigs	- Pigs that are not housed gain access to feces and eat.
	Maintaining a good relationship with the public	- When you buy a pig and find masese in after slaughter, the farmer won't believe it's his pig and will refuse to give the money back.
	Inadequate sanitation	- Some people don't have toilets.

	Undercooked pork	- In the past we used to just eat, even when it was just lightly roasted, even before it is fully cooked, we would just eat.
	Unavailability of drugs	- We are appealing that you ensure that drugs for masese become available here in Eastern province.
	Lack of knowledge: HCC/NCC	- If you eat pork infected with masese you will get seizures.
	Lack of knowledge: PCC	- I was happy to learn that there is actually medicine to give the pigs so that the mases can disappear.<br>- Elders said pigs got masese after being fed the remnants of beer brewing.<br>- They used to say that the cysts would go away if you would give the pig ashes in the mouth or to put ash in the maize bran before feeding.<br>- Sometimes they said that the cysts would go away when giving bicarbonate soda.
	Lack of knowledge: Relation PCC-HCC-taeniosis	- I didn't know the tapeworm comes from us and now I know that it's us who give pigs the masese.

Table 6: Behavioral change (Not what they will do but what they did since the educational workshop)

Group	Behavioral change	Specific comments
Lusaka 1	Checking for PCC in live pigs	- It's only now that we have started checking under the tongue.
	Teaching others	- I have taught at least 4 to 5 people since learning about the worm. I used what I learned, the knowledge I got from you, to teach them about masese.<br>- I told them that we learnt about the pork tapeworm and that people who have it can behave like this.
	Not selling infected pigs and/or pork	- Now when there is a pig with masese, it will stay without being bought from morning to afternoon.
	Sending a potential NCC patient to the hospital.	- I told them that maybe they should just take him to the hospital so they can test him for the worm because I think in their family, they have this problem.
	Thoroughly cooking pork.	- Now I tell my wife not to quickly roast it for me even when I am in a hurry.
Lusaka 2	Used the USB sticks	- Those flash drives you gave us, we used them on our computers.

Table 7: Extra

	Groups	Specific comments
Perceived PCC prevalence	PCC observed by all groups	- In all groups people had also seen pigs having seizures
Perceived prevalence of people having seizures	NCC observed by all groups	- L2: We have seen many people having seizures.
Confusion with ASF	Katete 1	- There are people like our parents who keep pigs and when these have seizures, they do not know what's

		happening so such videos will make them understand. Because they always say its ASF and yet it could be another disease.
Illiteracy	Katete 1	- Many people don’t even know how to read.
Ubiquity of pig rearing	Katete 2	- Almost everyone in the village has reared pigs.
