# Supplementary material for: Effects of ‘The Vicious Worm’ educational software on Taenia solium knowledge among key pork supply chain workers in Zambia
Source: PLoS Negl Trop Dis. 2020 Oct 19;14(10):e0008790. doi: 10.1371/journal.pntd.0008790 (PMC7595624; doi:10.1371/journal.pntd.0008790)
Supplement: S3 File — The checklist used to collect observational data. (PDF) [file pntd.0008790.s004.pdf]

Observation checklist

☐ Is meat inspection performed?

____

____

____

☐ What kind of inspection do they do?

____

____

____

☐ Do they hang up the pigs when they are killed to prevent contamination?

____

____

____

☐ Do they wash their hands between the different carcasses?

____

____

____

☐ Do they wash and disinfect their knives and other utensils used between different carcasses?

(e.g. in boiling water)

____

____

☐ Do they clean and disinfect the working surfaces between the different carcasses?

☐ Do they inspect all carcasses or a random selection?

☐ What kind of hygienic protection do they wear?

☐ Do you see any carcasses or parts of them that will be destroyed/ are being destroyed? Do you see a place/equipment for incineration?

☐ Does the slaughter place provide enough facility for the disposal of slaughter waste and wastewater?

☐ Are there people in the slaughterhouse that should not be there?

☐ Is there a system in place to track the meat?

☐ Is the slaughter area sealed off from the outside?

☐ Is there a cold chain? How is meat stored?

☐ Other observations? (Pest control, transport of meat, …)
