# Supplementary material for: Effects of ‘The Vicious Worm’ educational software on Taenia solium knowledge among key pork supply chain workers in Zambia
Source: PLoS Negl Trop Dis. 2020 Oct 19;14(10):e0008790. doi: 10.1371/journal.pntd.0008790 (PMC7595624; doi:10.1371/journal.pntd.0008790)
Supplement: Attachment — Submitted filename: Responses to review comments PNTD-D-20-01013 01092020.pdf [file pntd.0008790.s005.pdf]

Responses to review comments PNTD-D-20-01013 01092020

KEY REVIEW CRITERIA REQUIRED FOR ACCEPTANCE?

Reviewer #1:

C1. The objectives are clear.

C2. The study design only allows the short-term evaluation, which is mentioned in the manuscript but not in the abstract conclusions.
Response: Abstract conclusions section has been updated to address this.

C3. Population is clearly described.

C4. I am not in a position to comment on the statistics. No concerns about ethical or regulatory requirements.

Reviewer #2:

C1. One major concern I have with the manuscript is the reliance on written English (rather than written Nyanja-Chewa) for the questionnaires. This potentially contributes to misunderstandings.
Response: This was added to the limitations.

C2. Indeed, the authors themselves point out an example of potential language/translation issues: "A popular misbelief suggested that pigs acquire PCC after the consumption of beer brewing residues, which pigs were sometimes fed. This might be due to the use of the local terms masese, m'sokwe and mase to describe the white nodular cysticerci while these terms literally translate to 'beer dregs'." This made me less enthusiastic about the study's results.
Response: "Locally porcine cysticercosis is called with the terms masese, m'sokwe and mase, which literally translates to 'beer dreg' due to the resemblance of beer brewing residues with white nodular cysticerci." Was clarified in the discussion.

C3. Line 260: How much was the financial incentive for study participation?
Response: 150 ZMW (75 at the end of the workshop and 75 at the end of the follow-up session) was given to each participant, which converted to 12 USD. Now it converts to 8 USD.

RESULTS

Reviewer #1:

C1. OK.

Reviewer #2:

C1. Abstract Background section is disjointed
Response: The abstract background has been restructured.

C2. What is a “slaughterslab” worker? It would be helpful if the authors could expand upon what pig-related activities are performed at a slaughterslab (presumably killing the pigs, but any other activities?).
Response: Added “Slaughterslab workers include the professionals who are responsible for the pig slaughter, the meat cutting, the selling of pork and the management at the slaughter slab facility.” when slaughterslab workers are mentioned for the first time.

CONCLUSIONS

Reviewer #1:

C1. The conclusions in the abstract over-interprets the value of the results. It concludes that the results shows TVW to be effective... and should be considered for integration in T. solium control programs. The conclusion in the abstract needs to clarify that it shows to be effective in the short term, and could (instead of should) be considered for integration.
Response: Abstract conclusions section has been updated to address this.

C2. The limitations are reasonably presented.

Reviewer #2:

C1. OK.

EDITORIAL AND DATA PRESENTATION MODIFICATIONS:

Reviewer #1:

C1. No comments.

Reviewer #2:

C1. No comments.

Responses to review comments PNTD-D-20-01013 01092020

SUMMARY AND GENERAL COMMENTS

Reviewer #1:

C1. This paper is about the short-term effects of “The Vicious Worm” (TVW) educational software on Taenia solium knowledge among key pork supply chain workers in Zambia. The authors showed an improved knowledge 3 weeks after workshops with the software using a questionnaire and FDG.

C2. The manuscript is in general well written and clear.

C3. The main issue with the manuscript is that it over-interprets the value of the results in the abstract when it concludes that the results shows TVW to be effective... and should be considered for integration in T. solium control programs. The conclusion needs to clarify that it shows to be effective in the short term, and could be considered for integration.
Response: Abstract conclusions section has been updated to address this.

Abstract:

C4. Line 34: Health education is regarded important but not essential. Suggest change the word essential to important.
Response: The word ‘essential’ has been replaced by the word ‘important’.

C5. Lines 52-55: The conclusions seem to over-interpret the value of “The Vicious Worm”. Indeed, it seems that is a useful and promising tool, but it was evaluated only 3 weeks after its use, so it is too soon to evaluate its impact and recommend its integration. Suggest toning down the conclusion/significance as mentioned above.
Response: The section was updated to prevent overinterpretation.

Author summary:

C6. Line 60: The word chemotherapy needs to be reviewed. If it refers to humans, it should be Preventive chemotherapy (chemotherapy alone is usually referred to cancer treatment – preventive chemotherapy refers to treating populations at risk). If the authors refer to pigs, the treatment with oxfendazole (or similar interventions in animals) is classified as metaphylaxis or medication, not as chemotherapy. To avoid discussion and confusion, if the authors refer to pigs, I suggest using the word medication.
Response: The sentence has been changed to “Treatment of human taeniasis and intervention in pigs (vaccination and anthelmintic treatment) have been established as essential tools to achieve short-term control, but health education will be crucial to sustain long-term control.”

C7. Line 60: add pigs after vaccination, to clarify it refers to the vaccination of pigs and not people.
Response: Changed to “intervention in pigs (vaccination and anthelmintic treatment)”

Manuscript:

C8. Line 96: What do you mean with pig farming as a transitory activity?

Response: Households often start pig rearing to address financial issues or to serve pork during big celebrations (funerals, weddings, Christmas celebrations, initiation ceremonies) and is often stopped after African swine fever outbreaks or shortage of feed.

C9. Was any incentive given to the participants in the questionnaires or FDG?
Response: Yes: Each participant was given 150 ZMW was given to each participant, which was about 12 USD. Now it converts to 8 USD.

C10. Line 451: note that behavioural change in references 28 and 30 was in different groups of people, with different educational background which has an impact as mentioned in line 404. The authors imply that what they found in pork supply chain workers would/could be as described in references 28 and 30, however the very substantial differences in the subjects between the different studies does not permit this conclusion. Ref 28 relates to vets, extension officers, medical officers, etc (Ertel) while in this paper are pig traders, slab workers and butchers which have a different education level. Ref 30 refers to school children.
Response: The comparison was deleted due to the substantial differences in the subjects.

C11. Line 458: needs to clarify it is in the short term.
Response: Effective health education tool has been specified as effective short-term health education tool.

Reviewer #2:

C1. No comments.
